# Insulin-like growth factor-I inhibition with pasireotide decreases cell proliferation and increases apoptosis in pre-malignant lesions of the breast: a phase 1 proof of principle trial

**DOI:** 10.1186/s13058-014-0463-1

**Published:** 2014-11-11

**Authors:** Baljit Singh, Julia A Smith, Deborah M Axelrod, Pietro Ameri, Heather Levitt, Ann Danoff, Martin Lesser, Cristina de Angelis, Irineu Illa-Bochaca, Sara Lubitz, Daniel Huberman, Farbod Darvishian, David L Kleinberg

**Affiliations:** 1 Department of Pathology, New York University School of Medicine, New York, 10016 NY USA; 2 Department of Medicine, Division of Oncology, New York University School of Medicine, New York, 10016 NY USA; 3 Department of Surgery, New York University School of Medicine, New York, 10016 NY USA; 4 Department of Medicine, Division of Endocrinology and Bunnie Joan Sachs Laboratory, New York University School of Medicine, 550 First Avenue, New York, 10016 NY USA; 5 Department of Veterans Affairs Medical Center, New York, 10016 NY USA; 6 Department of Internal Medicine, University of Genoa, Genoa, 16132 Italy; 7 Feinstein Institute for Medical Research, North Shore - LIJ Health System, Manhasset, 11030 NY USA; 8 Rutgers Robert Wood Johnson Medical School, New Brunswick, 08901 NJ USA

## Abstract

**Introduction:**

Estrogen inhibition is effective in preventing breast cancer in only up to 50% of women with precancerous lesions and many experience side effects that are poorly tolerated. As insulin-like growth factor I (IGF-I) underlies both estrogen and progesterone actions and has other direct effects on mammary development and carcinogenesis, we hypothesized that IGF-I inhibition might provide a novel approach for breast cancer chemoprevention.

**Methods:**

In total, 13 women with core breast biopsies diagnostic of atypical hyperplasia (AH) were treated for 10 days with pasireotide, a somatostatin analog which uniquely inhibits IGF-I action in the mammary gland. They then had excision biopsies. 12 patients also had proliferative lesions and one a ductal carcinoma in situ (DCIS). Primary outcomes were changes in cell proliferation and apoptosis after treatment. Expression of estrogen receptor (ER), progesterone receptor (PR), and phosphorylated Insulin-like growth factor I receptor (IGF-1R), protein kinase B (AKT) and extracellular signal-regulated kinases 1/2 (ERK1/2) were also assessed. Core and excision biopsies from 14 untreated patients served as non-blinded controls. Hyperglycemia and other side effects were carefully monitored.

**Results:**

Pasireotide decreased proliferation and increased apoptosis in all AH (from 3.6 ± 2.6% to 1.3 ± 1.2% and from 0.3 ± 0.2% to 1.5 ± 1.6%, respectively) and proliferative lesions (from 3.8 ± 2.5% to 1.8 ± 1.8% and from 0.3 ± 0.2% to 1.3 ± 0.6%, respectively). The DCIS responded similarly. ER and PR were not affected by pasireotide, while IGF-1R, ERK1/2 and AKT phosphorylation decreased significantly. In contrast, tissue from untreated controls showed no change in cell proliferation or phosphorylation of IGF-1R, AKT or ERK 1/2. Mild to moderate hyperglycemia associated with reduced insulin levels was found. Glucose fell into the normal range after discontinuing treatment. Pasireotide was well tolerated and did not cause symptoms of estrogen deprivation.

**Conclusions:**

IGF-I inhibition by pasireotide, acting through the IGF-1R, was associated with decreased proliferation and increased apoptosis in pre-malignant breast lesions and one DCIS. Assuming hyperglycemia can be controlled, these data suggest that inhibiting the IGF-I pathway may prove an effective alternative for breast cancer chemoprevention.

**Trial registration:**

NCT01372644 Trial date: July 1, 2007.

**Electronic supplementary material:**

The online version of this article (doi:10.1186/s13058-014-0463-1) contains supplementary material, which is available to authorized users.

## Introduction

Breast cancer is one of the most frequently diagnosed tumors worldwide. Randomized controlled trials demonstrated the preventive effects of tamoxifen and raloxifene in women at increased risk [1]-[3]. These studies have led to the practice of employing selective estrogen receptor modulators (SERMs) for breast cancer chemoprevention. However, a number of side effects limit their use, including climacteric symptoms in premenopausal women, thromboembolic phenomena, and for tamoxifen, endometrial hyperplasia and carcinoma. The compliance rate with anti-estrogen therapy in the chemopreventive setting has been reported to be as low as 20% [4]. Breast cancer can be prevented in up to 50% of patients with high- risk lesions. Therefore, targeted estrogen inhibition is only partially effective. Alternative more effective medical approaches are highly desirable.

Insulin-like growth factor I (IGF-I) affects mammary development in at least two ways. It has an independent effect on ductal morphogenesis and is also essential for estrogen and progesterone action in the mammary gland [5]-[7]. Furthermore, IGF-I plays a pivotal role in the multi-step process that leads from normal breast tissue to hyperplasia and then carcinoma [8]. Based on these actions, we hypothesized that IGF-I inhibition would prevent breast cancer development. Indeed, published [9] and as yet unpublished data from our laboratory have shown that blockade of IGF-I action in the mammary gland prevents the development of premalignant mammary lesions in different mouse models [9]-[11]. In these experiments, we used a novel multi-ligand somatostatin analog, pasireotide, which not only inhibits growth hormone secretion from the pituitary and thereby, serum IGF-I, but also specifically targets IGF-I action within the mammary gland [12]. To translate the findings in rodents to humans, we conducted the present study, aimed at assessing the effect of IGF-I inhibition with pasireotide on premalignant lesions of the breast. Treatment with pasireotide caused a decrease in cell proliferation and an increase in apoptosis in all cases evaluated. Based on these results, we conclude that IGF-I inhibition may represent a novel strategy to prevent breast cancer.

## Methods

### Study setting

This study was conducted at the Clinical Cancer Center, the Clinical Translational Science Institute, the Immunohistochemistry and Histopathology Cores, and the BioRepository Center of the New York University (NYU) School of Medicine, and at the Department of Veterans Affairs Medical Center, New York, NY in accordance with the Helsinki Declaration. It was approved by the Institutional Review Board (IRB) at the NYU School of Medicine, the NYU Cancer Center Protocol Review and Monitoring Committee (PRMC) and the Department of Defense; Human Research Protection Office (HRPO), Office of Research Protections (ORP), United States Army Medical Research and Materiel Command (USAMRMC), the United States Food and Drug Administration (FDA), Novartis Pharmaceuticals and the Veterans Administration Office of Research and Development and Human Subjects Committee. The clinical trial number was NCT01372644.

### Participants

**Figure 1:**
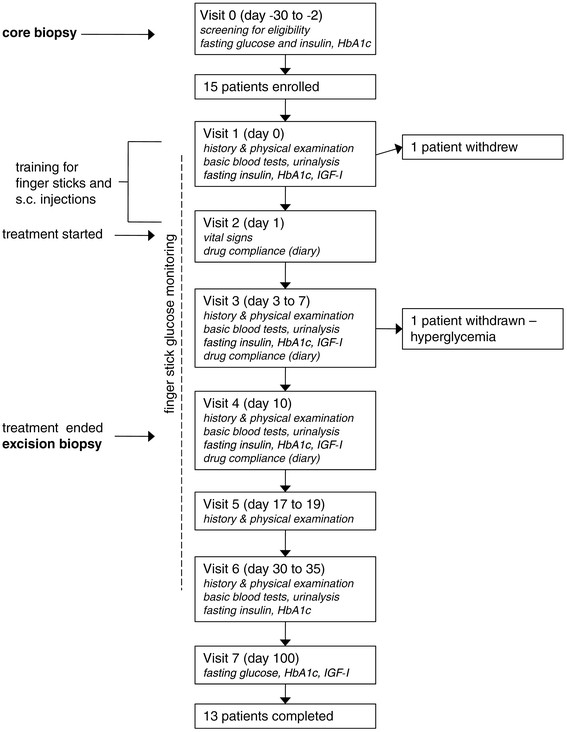
**Trial profile.** IGF, insulin-like growth factor; s.c., subcutaneous.

The trial included women who were diagnosed with atypical hyperplasia (AH) of the breast (atypical ductal hyperplasia (ADH) or atypical lobular hyperplasia/lobular carcinoma *in situ* (ALH/LCIS)) by core biopsy of lesions discovered by mammography (mass, density, calcifications). To be eligible, participants had to be over 21 years of age, be otherwise healthy, and be accessible for follow up. Patients in whom invasive breast cancer coexisted with AH or who had received tamoxifen or other anti-estrogens within the previous 6 months were excluded. Women with abnormal fasting plasma glucose or a QT interval longer than 450 msec were also excluded. Fifteen patients were enrolled and 13 completed the trial (Figure 1). Written informed consent was obtained from all.

### Procedures

IGF-I inhibition was achieved by treatment with 600 μg pasireotide (Novartis, Basel, Switzerland) subcutaneously injected twice daily for ten days. The patients self-injected the medication according to the instructions received at study entrance. They were also trained to measure finger-stick glucose with an Ascensia Contour^®^ glucometer (Bayer Health Care, Mishawaka, IN, USA) and to follow a low carbohydrate diet. The day before starting the treatment, participants underwent a baseline evaluation comprising a complete medical examination, basic blood tests, and measurement of fasting insulin, HbA1c, and IGF-I. During the 10-day treatment period, patients monitored fasting glucose by finger stick every day and also attended the study center between days 3 and 7 for additional safety assessment. Women who had fasting finger-stick glucose >120 mg/dL were asked to monitor glucose four times a day. On the tenth day they underwent standard-of-care excision biopsies with pre-operative wire placement. Women who had glucose levels ≥110 mg/dL at the end of treatment were asked to continue to monitor finger-stick glucose until glucose was normalized. Follow-up assessments were done 7 to 9 days, 20 to 25 days, and 3 months after the last injection of pasireotide (coinciding with the day of surgery) (Figure 1).

Both the core and excision biopsies were reviewed by a breast pathologist to confirm diagnosis. One patient who had ductal carcinoma in situ (DCIS) in addition to AH was inadvertently included in the trial. Twelve of thirteen patients also had various proliferative lesions (usual ductal hyperplasia, sclerosing adenosis, small ductal papilloma, radial scar). The effects of pasireotide on these lesions were also analyzed.

### Immunohistochemistry

Proliferation and apoptosis in epithelial cells were assessed by comparing formalin-fixed paraffin-embedded tissue sections of the core and excision biopsies. To reduce the possibility of chance effects, we also determined proliferation in core and excision biopsies from non-blinded patients who were not treated. Blocks were retrieved from the BioRepository Center of the NYU School of Medicine.

Proliferating cells were identified by immunohistochemistry for Ki67 [9]. Slides were incubated with Novocastra rabbit polyclonal anti-Ki67 antibodies (Leica Biosystems, Newcastle Upon Tyne, UK) and horseradish peroxidase (HRP)-conjugated goat anti-rabbit secondary antibodies (Dako, Carpinteria, CA, USA) and a 3,3’-diaminobenzidine (DAB) solution (Vector Laboratories, Burlingame, CA, USA) were used to detect the bound primary antibodies. The latter were omitted in negative controls. Apoptosis was demonstrated by terminal deoxynucleotidyl transferase (TdT) dUTP nick end labeling (TUNEL) [9]. This technique is based on the activity of the TdT enzyme, which labels the fragmented DNA of apoptotic cells for subsequent detection with a chromogenic reaction. In this study TUNEL was performed using the ApopTag^®^ kit (EMD Millipore, Billerica, MA, USA). Negative control tissue samples were not incubated with TdT.

The expression of estrogen receptor (ER) and progesterone receptor (PR) was also determined by immunohistochemistry with rabbit monoclonal primary antibodies (Thermo Fisher Scientific, Waltham, MA, USA). Biotinylated anti-rabbit secondary antibodies, followed by streptavidin-HRP conjugate and DAB, were used for detection. Stained sections were imaged on a light microscope with Nikon ACT-1 software. For each lesion, the percentage of Ki67-positive, TUNEL-positive, ER-positive, and PR-positive cells was counted out of a total of 200 epithelial cells.

After completing the immunostaining for Ki67, ER, and PR and the TUNEL, there was good-quality tissue left for further staining in five cases only for phosphorylated AKT and ERK 1/2 and four cases for phosphorylated IGF-1R in both core and excision biopsies. Sections were stained for phosphorylated AKT (Ser473) and phosphorylated ERK 1/2 (Thr202/Tyr204) by using rabbit monoclonal primary antibodies (Cell Signaling Technologies, Danvers, MA, USA) and biotinylated anti-rabbit secondary antibodies, streptavidin-HRP, and DAB for detection. Phosphorylation of AKT and ERK 1/2 was also assessed in the control samples from untreated patients. As the intensity of the immunostaining for phosphorylated AKT and ERK1/2 was heterogeneous, an intensity score (from 0 to 3) and a percentage score were ascertained for each lesion. A final score was then calculated by applying the following formula:

$$\left(\text{percentage}\;\mathrm{of}\;\text{weakly}\;\text{stained}\;\text{cells}\;*\;1\right)\;+\;\left(\mathrm{percentage}\;\text{of}\;\text{moderately}\;\text{stained}\;\text{cells}\;*\;2\right)\;+\;\left(\text{percentage}\;\mathrm{of}\;\text{strongly}\;\text{stained}\;\text{cells}\;*\;3\right)\text{.}$$

Immunofluorescence for phosphorylated IGF-1R was performed using rabbit anti p-IGF-1R (Y1161) (Abcam, Cambridge, MA, USA) as primary antibody and a secondary antibody obtained from Donkey anti rabbit Alexa 488 (Life technologies, Grand Island, NY, USA). To quantify the phosphorylated IGF-1R (Y1161) ten images per case, which included glandular tissue, were randomly imaged using 40× objective with 0.95 numerical aperture Zeiss Plan-Apochromat objective on a Zeiss Axionvert equipped with epifluorescence. Mean intensity was evaluated for each treatment on twelve-bit images using in-home developed macros for the open source platform Fiji-ImageJ (NIH, Bethesda, MD, USA).

### Statistical analysis

Descriptive statistics are presented as mean and SD and changes from before (core biopsy) to after (excision biopsy) treatment as both the actual arithmetic difference and the percent change relative to pre-treatment. Relative percent change is the preferred statistic, because it is adjusted for the before-biopsy value (for example, a decrease in Ki-67 from 5% to 4% is a 1% arithmetic decrease, but a 20% relative decrease). The primary endpoints were the changes and relative changes in cell proliferation and apoptosis from the core to the excision biopsies. The evaluation of ER and PR status before and after treatment was the secondary pre-specified endpoint.

Due to the small sample size, a non-parametric approach was taken and the Wilcoxon signed rank (WSR) test was used to analyze the study outcomes. The sign test was also used to determine whether directionality of change differed from chance. Analysis was conducted separately on proliferative lesions and AH lesions. Two pathologists blinded to patient status read the slides for Ki67 and apoptosis. After that, one left the institution and the remaining slides (PR, ER, phosphorylated AKT and ERK 1/2) were analyzed only by the other pathologist. The paired *t*-test and repeated-measures analysis of variance (ANOVA) were used to compare the scores for phosphorylated AKT and ERK 1/2 before and after treatment and pIGF-1R by fluorescence. Blood concentrations of glucose, insulin, and IGF-I across study visits, respectively were also analyzed. In a separate sub-study, we demonstrated excellent inter-rater agreement between two pathologists who, blinded to one another, evaluated Ki-67 in 12 treated patients and 23 controls.

### Role of the funding source

Neither the funding source nor the provider of the medication had any role in the design or conduct of the study, or in the collection, analysis, and interpretation of the data. The corresponding author had full access to all data and had final responsibility for the decision to submit for publication.

## Results

### Patients

All patients were Caucasian. Mean age was 55.9 ± 6.2 years. Four women had ADH, three had a combination of ADH and ALH/LCIS, five had ALH/LCIS, and one had ALH/LCIS and DCIS. Some AH lesions were entirely removed with the core biopsy. However, those cases had remaining areas of proliferative disease in both core and excision biopsies that permitted evaluation of the effects of pasireotide. In all, there were twelve proliferative lesions, five ALH/LCIS, one ADH, and one DCIS available for assessment of the study endpoints. Control samples from untreated patients included ten proliferative and five AH lesions from 14 women whose mean age was 49.8 ± 6.9 years.

### Effect on cell proliferation

**Figure 2:**
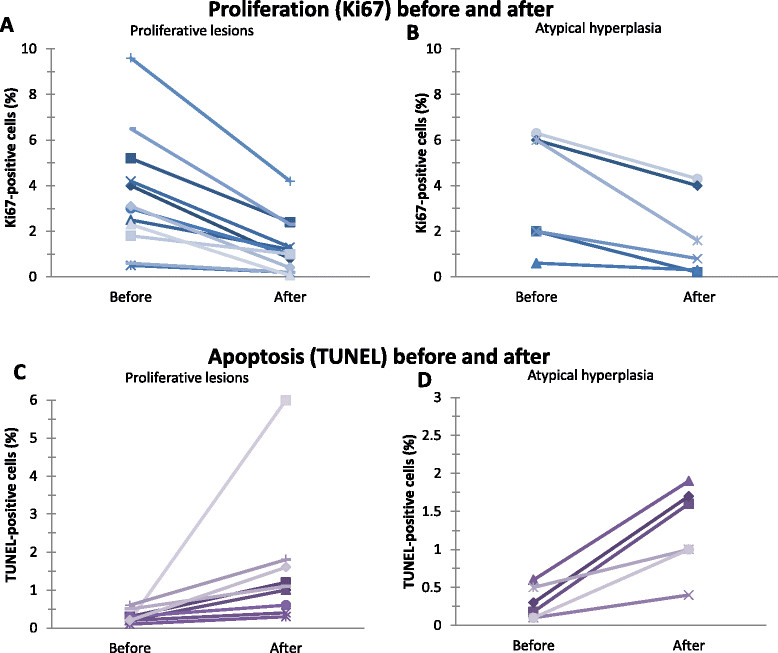
**Effect of treatment with pasireotide on cell proliferation and apoptosis in pre-malignant lesions of the breast.** Effects of pasireotide on proliferation rate as assessed by immunohistochemistry for Ki67 in 12 proliferative **(A)** and 6 atypical hyperplasia **(B)** Apoptosis, as assessed by terminal deoxynucleotidyl transferase dUTP nick end labeling (TUNEL), in 11 proliferative **(C)** and 6 atypical hyperplasia **(D)** lesions of the breast before and after 10 days of treatment with pasireotide.

**Figure 3:**
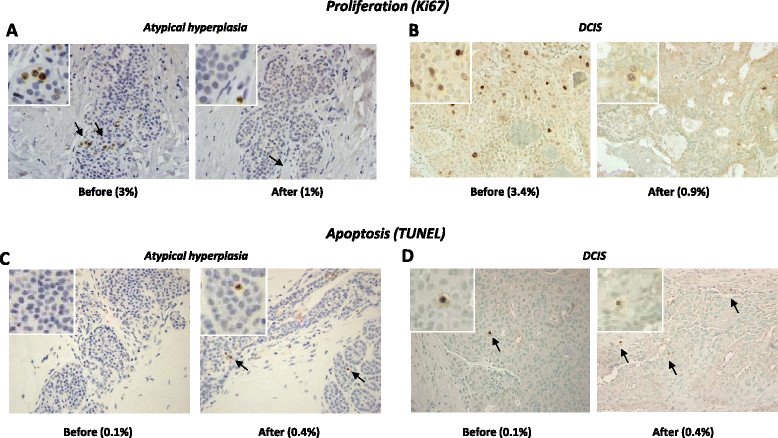
**Representative images of proliferation (Ki67) and apoptosis (terminal deoxynucleotidyl transferase dUTP nick end labeling (TUNEL)) from patients treated with pasireotide.** Representative examples showing Ki67 immunostaining of atypical hyperplasia **(A)** and one case of ductal carcinoma *in situ* (DCIS) **(B)** from pasireotide-treated patients (top panel). **(C)** TUNEL staining of an atypical lobular hyperplasia (ALH) lesion before and after treatment with pasireotide. Positive cells are brown. **(D)** A small increase in the number of apoptotic cells (arrows) was also observed after treatment in one case of DCIS.

Compared with pre-treatment core biopsies, pasireotide caused a reduction in cell proliferation in all proliferative and AH lesions in the excision biopsies (WSR, *P* = 0.001 and *P* = 0.031 respectively) (Figure 2A). On average, Ki67-positive cells decreased by 66.5% in proliferative lesions and by 56.7% in areas of AH. Cell proliferation also decreased by 73.5% (from 3.4% to 0.9%) in the DCIS lesion (Figure 3B).

The percentage of Ki67-positive cells decreased in six of the ten control proliferative lesions (sign test, *P* = 0.29) and overall it did not significantly vary between the core and excision biopsies (mean change -1.2 ± 7.5%; WSR, *P* = 0.54). Similarly a reduction in proliferation was observed in two out of five AH lesions from untreated patients (Figure 2B). These decreases were not significantly different from the hypothesis of random change (sign test, *P* = 1.00 and *P* = 0.77, respectively), nor was the mean percentage of Ki67-positive cells significantly different in core versus excision biopsies (1.1 ± 5.2%, WSR *P* = 0.44 for AH and -0.6 ± 16.1%, WSR *P* = 0.97 for DCIS), (Figure 3C-D). These results represent the work of pathologist BS.

### Effect on apoptosis

**Figure 4:**
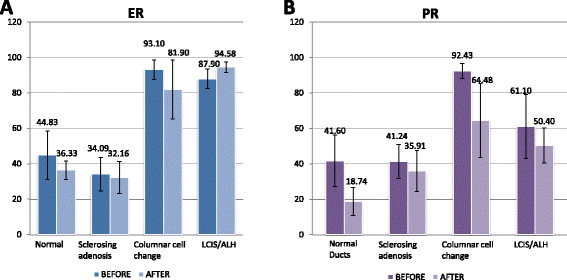
**Effect of pasireotide on the estrogen and progesterone receptor.** No significant differences were observed in the levels of estrogen receptor (ER) **(A)** or progesterone receptor (PR) **(B)** before and after treating with pasireotide.

An increase in apoptosis was found in all proliferative and AH lesions upon treatment with pasireotide (WSR, *P* = 0.001 and WSR, *P* = 0.031, respectively; Figure 4A-C), as well as in the DCIS (from 0.1% to 0.4%; Figure 4D).

### Effects on ER and PR

Pasireotide had no significant effect on ER or PR. Effects of pasireotide on ER and PR are shown in Table 1 and Figure 4.

**Table 1:** **Changes in the expression of estrogen receptor (ER) and progesterone receptor (PR) following treatment with pasireotide**

	Number	Change in percentage of positive cells	*P* -value	Number of lesions in which the percentage of positive cells decreased	*P* -value
**Proliferative lesions**					
ER	9	–4.2 ± 9.9	0.13	7 (77.8%)	0.18
PR	7	–9.8 ± 30.7	0.81	3 (42.9%)	1.00
**Atypical hyperplasia**					
ER	5	–8.0 ± 15.0	0.31	2 (40%)	1.00
PR	4	–10.7 ± 54.3	0.88	2 (50%)	1.00

Differences in the percentage of positive cells in core versus excision biopsies are presented as mean ± SD.

### Mechanism of action of pasireotide

**Figure 5:**
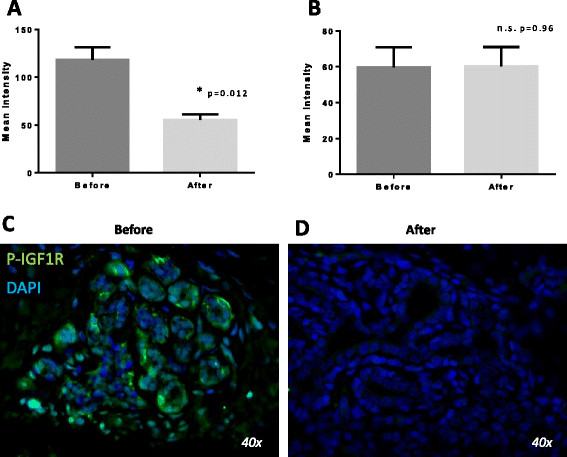
**Effect of treatment with pasireotide on phospho insulin-like growth factor -1 receptor (IGF-1R).** Effect of pasireotide on mean intensity of phospho IGF-1R by immunofluorescence in four patients with atypical hyperplasia before and after treatment with pasireotide **(A)** and five controls **(B)**. Representative sections from one of the patients before **(C)** and after treatment **(D)**. DAPI: 4',6-diamidino-2-phenylindole; n.s., not significant.

We recently reported that pasireotide inhibited cell proliferation and increased apoptosis in a model of rat mammary hyperplasia induced by hGH + E_2_ by blockade of the IGF-I receptor, as measured by a reduction in pIRS1 [9]. To determine if pasireotide also worked through that mechanism in women with atypical hyperplasia we measured the effect of pasireotide on phospho IGF-1R expression, as measured by intensity of immunofluorescence, and found that it was significantly lowered in excision biopsies after treatment compared to pre-treatment core biopsies. Measurements in before and after core and excision biopsies in untreated patients served as controls (Figure 5).

**Figure 6:**
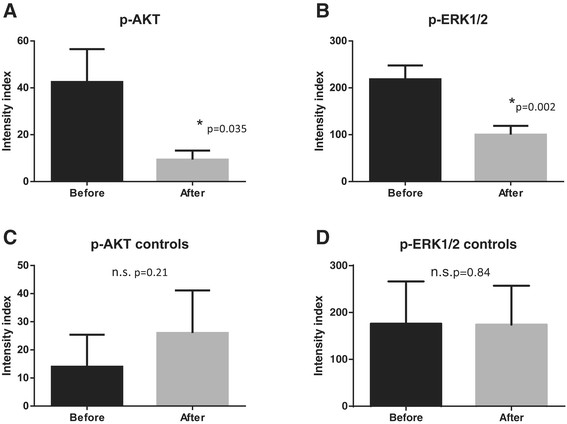
**Effect of treatment with pasireotide on phospho AKT and ERK1/2. (A)** The mean intensity index was decreased by pasireotide from 42.5 ± 14 to 9.37 ± 3 after treatment as measured in eight patients. **(B)** Similar effects were observed with phospho ERK1/2 with a mean intensity index of 218 ± 29 before to 100 ± 18 after treatment, measured in six patients. Control tissues were not affected for p-AKT **(C)** or p-ERK 1/2 **(D)**.

Phosphorylated AKT and ERK 1/2 were also affected in five proliferative, two ALH/LCIS, and one ADH from five women. Both the number of positive cells and the intensity of the staining were lower in excision than in core biopsies (Figure 6).

### Effects on glucose, insulin, HgbA1c and IGF-I and side effects

**Figure 7:**
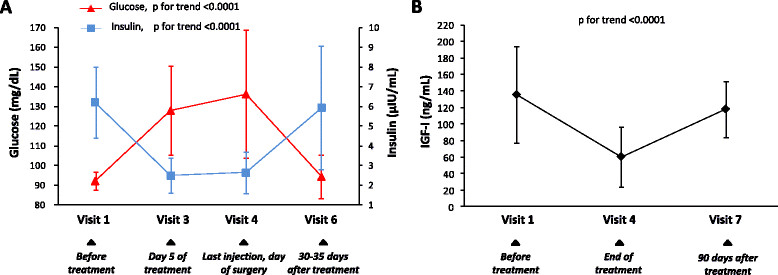
**Effects of treatment with pasireotide on glucose, insulin, and insulin-like growth factor -1 (IGF-I). (A)** Mean concentrations of plasma glucose and serum insulin during the 10-day treatment with pasireotide and the subsequent follow up. **(B)** Mean serum concentration of IGF-I before, at the end of treatment, and 90 days after treatment with pasireotide. Error bars are SDs in both graphs.

Among the 14 treated patients, 11 (78.6%) had grade 1 nausea and seven (50%) grade 1 diarrhea. Both adverse events were transient. Side effects of estrogen inhibitors, such as hot flashes, night sweats, decreased libido, and vaginal dryness, were not reported. Mean fasting finger-stick glucose rose from the baseline value of 97.8 ± 14.2 mg/dL to a peak of 133.2 ± 23.9 mg/dL (Figure 7). On average the highest glucose value was reached seven days after the first injection of pasireotide. One patient was withdrawn from the study after her postprandial capillary glycemia reached 226 mg/dL. Four out of the thirteen study participants had fasting finger-stick glucose values ≥110 mg/dL on the last day of treatment. Their mean glucose decreased from 162.8 ± 21.2 mg/dL to 100.5 ± 18.8 mg/dL in one day, and by three days after drug discontinuation it was 92.0 ± 16.2 mg/dL. An additional five patients also continued to monitor glucose after day 10. Overall, fasting glucose was 98.7 ± 13.4 mg/dL by day 13.

Hyperglycemia was associated with a reduction in insulin levels, which returned to normal by day 30 to 35 (Figure 7). HbA1c concentrations were not affected by treatment (5.6 ± 0.4% at baseline and 5.6 ± 0.3% three months after stopping the drug). Serum IGF-I decreased in all patients, confirming adherence to treatment, although it remained within the age-specific normal range in 11 (84.6%). It returned to normal by three months (Figure 5B).

## Discussion

In recent years a number of IGF-I inhibitors have been tested in the treatment of various carcinomas. Both antibodies to the IGF-I receptor and small molecule inhibitors of its tyrosine kinase activity have been employed, usually together with other anti-cancer drugs [13]-[17]. Efficacy has been mixed, at best. The results led Yee to liken IGF-I inhibition to `the baby or the bathwater’ [18]. Those studies were largely carried out in patients with advanced solid tumors.

We have approached IGF-I inhibition from a different vantage point. Studies from our laboratory show that growth hormone-induced IGF-I is essential for mammary development in mice and rats [6],[7], and that growth hormone (GH), or IGF-I absence or inhibition, block development [12],[19]. In addition, administration of GH plus physiological concentrations of estradiol was found to cause mammary hyperplasia in hypophysectomised, oophorectomized rats [9] and pasireotide prevented it. Pasireotide is uniquely effective in inhibiting the action of IGF-I in the mammary gland. Like the older somatostatin analogs, octreotide and lanreotide, it reduces GH secretion from the pituitary gland, which results in decreased liver expression of IGF-I, and thereby reduced serum levels. By virtue of its broader somatostatin receptor affinity profile, however, pasireotide also potently inhibits IGF-I activity directly in the mammary gland, likely by stimulating the expression of IGF-I binding protein 5, which preferentially binds to IGF-I making it unavailable for its receptor [12]. Although a side-by-side comparison of pasireotide with tamoxifen was not done, the experiments provided evidence that pasireotide was at least as, or possibly more, effective than tamoxifen in preventing hormone-induced hyperplasia [9]. It should be pointed out that we did not measure IGF-2 because specimens became unavailable.

Anti-estrogen chemoprevention in patients with atypical lesions significantly decreases development of carcinoma [2]. In this study we tested IGF-I inhibition for breast cancer prevention in humans, by analyzing the effect of pasireotide on different types of precancerous lesions including proliferative lesions, atypical hyperplasia and also one case of ductal carcinoma *in situ*. Atypical hyperplasias are characterized by genetic alterations and gene expression profiles similar to those of invasive carcinomas [20] and are associated with an increased risk for developing cancer [21]. We postulated that earlier lesions such as atypical hyperplasia would maintain their responsiveness to IGF-I inhibition. This proof-of-principal trial used surrogate biological endpoints (proliferation and apoptosis) in patients with atypical breast lesions to test the efficacy of IGF-I inhibition. Indeed, pasireotide significantly decreased cell proliferation in proliferative lesions and AH during 10 days of treatment. Importantly, pasireotide also reduced the percentage of proliferating cells in one case of DCIS.

Phase 1 studies typically do not include randomized placebo-controlled arms. To help confirm observations in the treated group we examined core and excision biopsies from 15 women with precancerous lesions of proliferative disease and atypical hyperplasia, who underwent excision biopsies as standard of care. They did not receive pasireotide between core and excision biopsies. There was no significant difference in proliferation from the hypothesis of random change between core and excision biopsies. Although the cell proliferation indices were low in both treated and untreated patients, the lack of a reduction in cell proliferation in the untreated patients in excision biopsies helps support the Ki67 results. Deregulated proliferation underlies the entire spectrum of precancerous breast lesions and it is also a main feature of established cancer [22]. Therefore, IGF-I inhibition by means of pasireotide affected a key event in breast carcinogenesis. Decreased proliferation is also thought to be a main mechanism by which estrogen inhibitors may prevent the development and inhibit the progression of breast cancer. Although the effects of pasireotide and estrogen inhibitors on cell proliferation have not been compared directly, the magnitude of change in published reports suggests that the effect of estrogen inhibitors is similar to that of pasireotide [23]-[26].

Recent reports indicate that analysis of ki67 can be highly variable between different readers and at different institutions [27]. To determine whether our pathology collaborators also experienced reading variability, we had two of them (BS and FS) independently read before- and after-treatment slides for cell proliferation in 10 patients (mean age 54.8 ± 4.7 years) and 23 untreated controls (mean age: 54.5 ± 9.1 years). The inter-rater agreement was excellent,

Pasireotide was also found to significantly increase apoptosis in proliferative lesions, AH, and in one case of DCIS. Apoptosis is particularly effective in eliminating aberrant cells which might play a role in cancer prevention. This effect may be secondary to the inhibition of IGF-I, which is anti-apoptotic [8], but also to an IGF-I-independent activity of pasireotide, as activation of the somatostatin receptors has been shown to directly induce cell cycle arrest and apoptosis in other tissues [28]. Tamoxifen is also pro-apoptotic in breast cancer cells, as well as in experimental tumors in rats [29],[30]. However, the effects of tamoxifen on apoptosis in women in prevention trials are not as well-documented, making comparison difficult [25],[31].

These studies show that pasireotide directly blocks the IGF-I receptor activity, which in turn, prevents free IGF-I binding to the IGF-1R [12]. It also increases IGF binding protein 5, which is thought to preferentially bind to local IGF-I more than to the IGF-1R [9]. Whether this event occurs after blockade of the IGF-1R or is independent is not known. That reduction in phosphorylated AKT and ERK 1/2, which are main intracellular mediators of IGF-I downstream IGF-I pathway, may explain the decrease in cell proliferation and increase in apoptosis [8],[32]. Specific inhibitors of AKT and ERK are presently being evaluated for treatment of breast cancer [32].

Pasireotide is presently approved for treatment of Cushing’s disease with the caveat that it can cause hyperglycemia or diabetes, or worsen existing diabetes [33]. The present trial provides evidence that the hyperglycemia of pasireotide is caused by a reduction in serum insulin, and another recent study found that the reduction of insulin is accompanied by a reduction in glucagon-like peptide-1 (GLP-1) and glucose-dependent insulinotropic peptide (GIP) [34]. These effects are clearly more problematic in patients with Cushing’s disease or women with underlying diabetes than they would be in otherwise normal individuals.

Minimal to moderate hyperglycemia would not pose a major risk to these patients during relatively short periods. In contrast, prolonged exposure might do so. Therefore, it would be important to prevent or lessen the impact of pasireotide on hyperglycemia. Possible approaches might include, altering the dose of pasireotide, adding glucagon-like peptide 1 agonists, or metformin [35], and/or using pasireotide intermittently. Preclinical studies in *Brca*1-deficient mice have shown that intermittent administration of pasireotide maintains its inhibitory effect on cell proliferation for at least 3 weeks after discontinuing medication (as yet unpublished). Our trial provides evidence that hyperglycemia disappears rapidly on stopping pasireotide. Thus, hyperglycemia might be decreased by reducing exposure to medication. Pasireotide is also available in a once-a-month preparation. However, until further studies are carried out, and a means of dealing with hyperglycemia is established, it is premature to suggest the use of pasireotide for breast cancer prevention.

## Conclusion

In conclusion, these results provide proof of principle that inhibiting the effect of IGF-I downstream of the IGF-I pathway with pasireotide causes a decrease in cell proliferation and an increase in apoptosis in precancerous breast lesions. Further trials might support the concept that IGF-I inhibition might serve as a chemoprevention tool for breast cancer, especially in patients unable to tolerate direct estrogen inhibition.

## Authors’ contributions

BS helped conceive and design the study, read and analyzed pathology slides, worked with fellows and scientists, and participated in writing the manuscript. JAS and DMA helped conceive and design the study, played major roles in clinical and intellectual aspects of the study, recruited and oversaw treatment, safety and participation of patients, and critically reviewed and revised the manuscript. PA performed experiments, carried out and analyzed biochemical endpoints, helped draft the manuscript, and prepared tables and graphs. HL coordinated patient participation and organized fellows and clinicians throughout the study, obtained IND from the FDA, coordinated regulatory document submissions, helped with preparation of required technical and annual progress reports, and critically read and participated in the writing the manuscript. AD participated in design and writing of the study protocol, oversaw evaluation of patients’ hyperglycemia and other side effects, and critically reviewed and helped prepare the manuscript. ML performed statistical analysis for all aspects of the clinical trial, and prepared the statistical analysis section of the manuscript. CdA performed staining, prepared and maintained records of archived slides and prepared standards for blind analysis of slides, and prepared graphs and tables for the manuscript. II-B performed analysis of effects of pasireotide on phosphorylated IGF-I receptor, prepared slides and conceived the semiquantitative analysis for phosphorylation of AKT and ERK 1/2, and prepared tables, graphs and figures for the manuscript. SL monitored patients daily during and after the study for compliance and safety, analyzed the data, prepared regulatory documentation for submission to various ethics committees and helped draft and critically review the manuscript. DH analyzed data relating to glucose, insulin and IGF-I and prepared graphs and tables included in the manuscript, and critically reviewed it. FD joined the study group to determine agreement between pathologists on reading Ki67, and critically reviewed the manuscript, and helped revise the pathology section. DLK, Principal Investigator, conceived the project and was responsible for the accuracy and integrity of data and for patient safety, and oversaw all aspects of the study including drafting the grant, protocol, and manuscript. All authors have read and approved the final version of this manuscript.

## Authors’ original submitted files for images

Below are the links to the authors’ original submitted files for images. Authors’ original file for figure 1 Authors’ original file for figure 2 Authors’ original file for figure 3 Authors’ original file for figure 4 Authors’ original file for figure 5 Authors’ original file for figure 6 Authors’ original file for figure 7

## Abbreviations

ADH: atypical ductal hyperplasia
AH: atypical hyperplasia
ALH: atypical lobular hyperplasia
ANOVA: analysis of variance
*Brca*1: breast cancer suppressor gene called breast cancer 1, early onset
DAB: 3, 3׳-diaminobenzidine
DCIS: ductal carcinoma *in situ*
dUTP: deoxyuridine triphosphate
E_2_: estrogen
ER: estrogen receptor
FDA: Food and Drug Administration
GH/hGH: growth hormone/human growth hormone
GIP: glucose-dependent insulinotropic peptide
GLP-1: glucagon-like peptide-1
HbA1c: hemoglobin A1c
HRP: horseradish peroxidase
HRPO: Human Research Protection Office
IGF: insulin-like growth factor
IGF-IR: insulin-like growth factor I receptor
IND: investigational new drug
IRB: Institutional Review Board
Ki67: immunostain to identify cell proliferation
LCIS: lobular carcinoma *in situ*
NIH: National Institutes of Health
NYU: New York University
ORP: Office of Research Protections
p-IGF-1R: phosphorylated IGF-I receptor
pIRS1: phosphorylated insulin receptor substrate
PR: progesterone receptor
PRMC: Protocol Review and Monitoring Committee
SERM: selective estrogen receptor modulator
TdT: terminal deoxynucleotidyl transferase
USAMRMC: United States Army Medical Research and Materiel Command
TUNEL: apoptosis
WSR: Wilcoxon signed rank
