# Supplementary figures and images for: Insulin-like growth factor-I inhibition with pasireotide decreases cell proliferation and increases apoptosis in pre-malignant lesions of the breast: a phase 1 proof of principle trial
Source: Breast Cancer Res. 2014 Nov 11;16:463. doi: 10.1186/s13058-014-0463-1 (PMC4303192; doi:10.1186/s13058-014-0463-1)

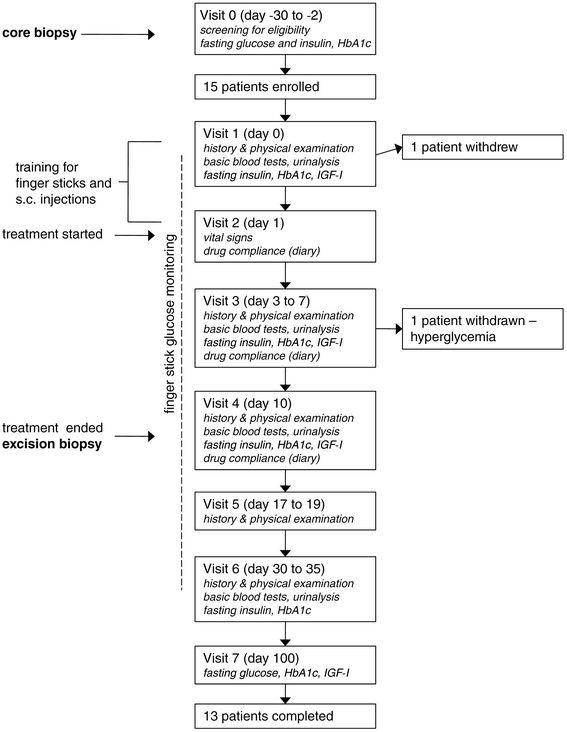

Supplement: Supplementary file 1 — Authors’ original file for figure 1 [file 13058_2014_463_MOESM1_ESM.gif]

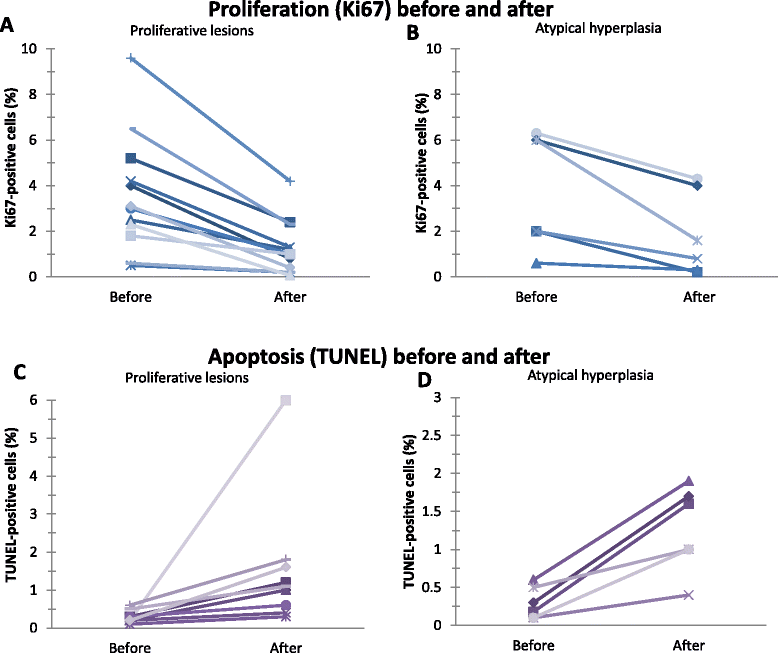

Supplement: Supplementary file 2 — Authors’ original file for figure 2 [file 13058_2014_463_MOESM2_ESM.gif]

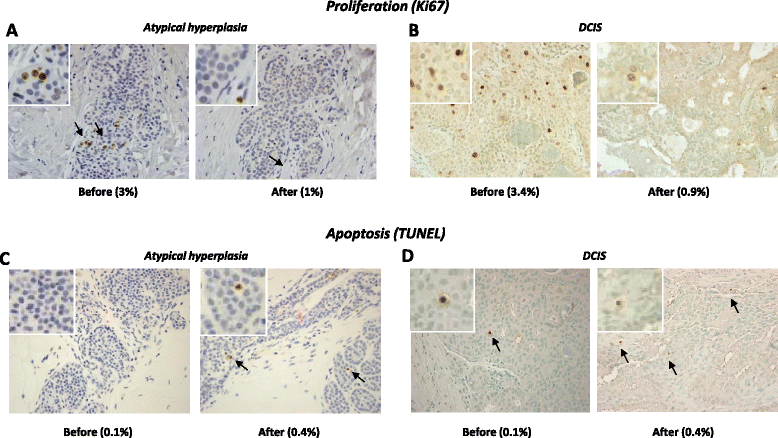

Supplement: Supplementary file 3 — Authors’ original file for figure 3 [file 13058_2014_463_MOESM3_ESM.gif]

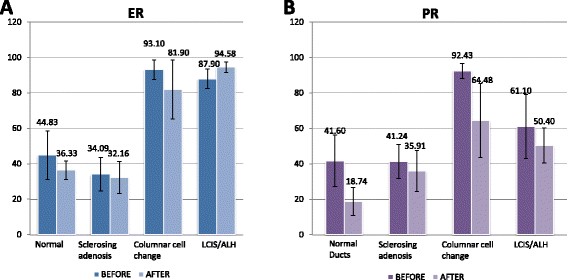

Supplement: Supplementary file 4 — Authors’ original file for figure 4 [file 13058_2014_463_MOESM4_ESM.gif]

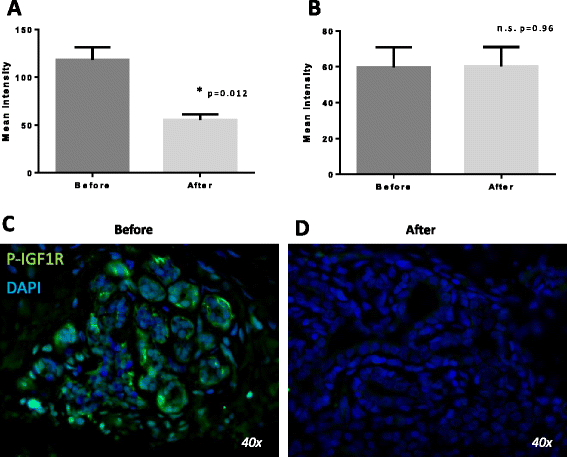

Supplement: Supplementary file 5 — Authors’ original file for figure 5 [file 13058_2014_463_MOESM5_ESM.gif]

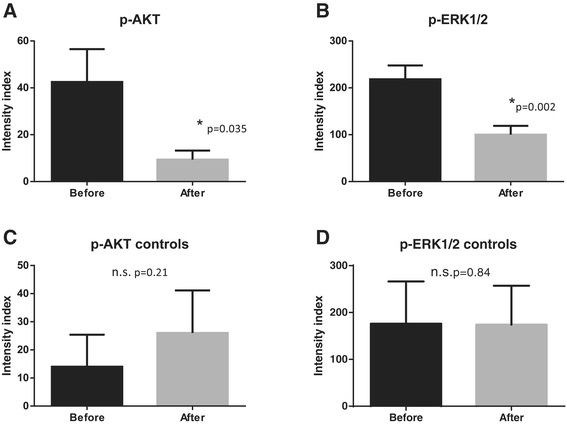

Supplement: Supplementary file 6 — Authors’ original file for figure 6 [file 13058_2014_463_MOESM6_ESM.gif]

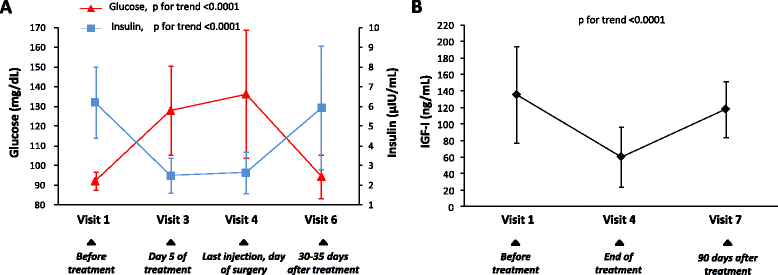

Supplement: Supplementary file 7 — Authors’ original file for figure 7 [file 13058_2014_463_MOESM7_ESM.gif]
